# A single-cell atlas of normal and KRAS^G12D^-malformed lymphatic vessels

**DOI:** 10.1172/jci.insight.185181

**Published:** 2025-01-28

**Authors:** Lorenzo M. Fernandes, Danielle Griswold-Wheeler, Jeffrey D. Tresemer, Angelica Vallejo, Neda Vishlaghi, Benjamin Levi, Abigail Shapiro, Joshua P. Scallan, Michael T. Dellinger

**Affiliations:** 1 Hamon Center for Therapeutic Oncology Research, and; 2 Department of Surgery, UT Southwestern Medical Center, Dallas, Texas, USA.; 3 Department of Molecular Pharmacology and Physiology, Morsani College of Medicine, University of South Florida, Tampa, Florida, USA.

**Keywords:** Angiogenesis, Development, Vascular biology, Endothelial cells

## Abstract

Somatic activating mutations in *KRAS* can cause complex lymphatic anomalies (CLAs). However, the specific processes that drive *KRAS*-mediated CLAs have yet to be fully elucidated. Here, we used single-cell RNA sequencing to construct an atlas of normal and Kras^G12D^-malformed lymphatic vessels. We identified 6 subtypes of lymphatic endothelial cells (LECs) in the lungs of adult wild-type mice (Ptx3, capillary, collecting, valve, mixed, and proliferating). To determine when the LEC subtypes were specified during development, we integrated our data with data from 4 stages of development. We found that proliferating and Ptx3 LECs were prevalent during early lymphatic development and that collecting and valve LECs emerged later in development. Additionally, we discovered that the proportion of Ptx3 LECs decreased as the lymphatic network matured but remained high in Kras^G12D^ mice. We also observed that the proportion of collecting and valve LECs was lower in Kras^G12D^ mice than in wild-type mice. Last, we found that immature lymphatic vessels in young mice were more sensitive to the pathologic effects of Kras^G12D^ than mature lymphatic vessels in older mice. Together, our results expand the current model for the development of the lymphatic system and suggest that KRAS mutations impair the maturation of lymphatic vessels.

## Abstract 2

Activating *KRAS* mutations can cause complex lymphatic anomalies. Using single-cell RNA sequencing, we show that hyperactive KRAS signaling impairs the maturation of lymphatic vessels.

## Introduction

Complex lymphatic anomalies (CLAs) are rare, life-threatening diseases caused by the abnormal development of lymphatic vessels (1). Gorham-Stout disease, generalized lymphatic anomaly, kaposiform lymphangiomatosis, and central conducting lymphatic anomaly fall under the umbrella of CLAs (1). Patients with CLA exhibit a range of phenotypes, including multifocal lymphatic malformations (LMs), tortuous dilated central lymphatic vessels, and ectopic lymphatic vessels in bone (2–7). Depending on the location of their disease, patients with CLA experience various complications. These complications include protein-losing enteropathy, lymphedema, chylous ascites, chylothorax, and bone loss (2–7). CLAs are diagnosed based on the patient’s medical history and findings from physical examination, imaging, and histology. More recently, investigators have attempted to diagnose patients based on genetic results. Next-generation sequencing of affected tissue, cell-free DNA, and lymphatic endothelial cells (LECs) from patients revealed that sporadically occurring CLAs can be caused by mutations in oncogenes (8–15). To date, somatic activating mutations in *PIK3CA*, *ARAF*, *BRAF*, *MAP2K1*, *HRAS*, and *NRAS* have been found in patients with CLA (8–15). However, no clear genotype-phenotype associations exist for CLAs (8, 13). The reason the same genetic mutation causes different CLAs is unknown, but could be due to when the mutation occurs during development, where it appears in the body, or the type of LEC harboring the genetic mutation (e.g., lymphatic endothelial progenitor versus other LEC subtypes).

We and others recently identified somatic activating mutations in *KRAS* in patients with CLA (5, 8, 14, 16–18). KRAS is a small GTPase that switches between inactive GDP-bound and active GTP-bound forms (19). GTP-bound KRAS promotes cell proliferation, migration, and survival by activating pathways such as the phosphatidylinositol 3-kinase (PI3K) and mitogen-activated protein kinase (MAPK) pathways (19). The KRAS mutations in patients with CLA increase KRAS signaling by impairing GTP hydrolysis or increasing GDP/GTP nucleotide exchange (19). Experiments with zebrafish embryos revealed that hyperactive KRAS signaling in LECs causes edema and the enlargement of lymphatic vessels (18). We recently showed that mouse embryos that express an active form of KRAS (KRAS^G12D^) in their LECs also have edema and enlarged lymphatic vessels (20). Additionally, we found that KRAS^G12D^ expression in LECs postnatally stimulates the formation of lymphatics in bone and impairs the formation of lymphatic valves (17). Transcriptional profiling of LECs revealed that KRAS^G12D^ decreases the expression of genes that promote lymphatic valve development (20). Importantly, trametinib (an FDA-approved MEK1/2 inhibitor) increases the expression of lymphatic valve genes in KRAS^G12D^ LECs and suppresses the loss of lymphatic valves in KRAS^G12D^ mice (17, 20). MEK1/2 inhibition also decreases the incidence of edema in zebrafish embryos (18). Despite these advances, the cellular and molecular mechanisms underlying how oncogenic KRAS mutations cause CLAs and their impact on LEC heterogeneity are not fully understood.

Single-cell RNA sequencing (scRNA-Seq) has emerged as a powerful tool to study the heterogeneity of cell populations and has significantly contributed to our understanding of LEC biology (21–24). Recent studies using scRNA-Seq have revealed a remarkable degree of heterogeneity within LECs, highlighting the existence of distinct subpopulations with diverse functions and gene expression profiles (21–24). Here, we use scRNA-Seq to study LEC heterogeneity and identify the impact of Kras^G12D^ on the cellular and transcriptional landscape of lymphatic vessels. We show that there are 6 subtypes of lymphatic endothelium in the lungs of normal mice (proliferating, Ptx3, capillary, collecting, valve, and mixed). We then characterize when the different LEC subtypes appear during development. We show that proliferating and Ptx3 LECs are prevalent during early lymphatic development and that capillary, collecting, and valve LECs emerge later in development. Additionally, we demonstrate that the proportion of Ptx3 LECs decreases as the lymphatic network matures, but remains high in Kras^G12D^ mice. We also show that Kras^G12D^ mice have fewer collecting and valve LECs than control mice. Last, we demonstrate that immature lymphatic vessels in young mice are more susceptible to the adverse effects of Kras^G12D^ than mature lymphatic vessels in older mice. Together, our results refine the current model for the development of the lymphatic system and suggest that hyperactive KRAS signaling suppresses the remodeling and maturation of lymphatic vessels.

## Results

### Kras^G12D^ impairs the development of pulmonary lymphatic vessels.

**Figure 1:**
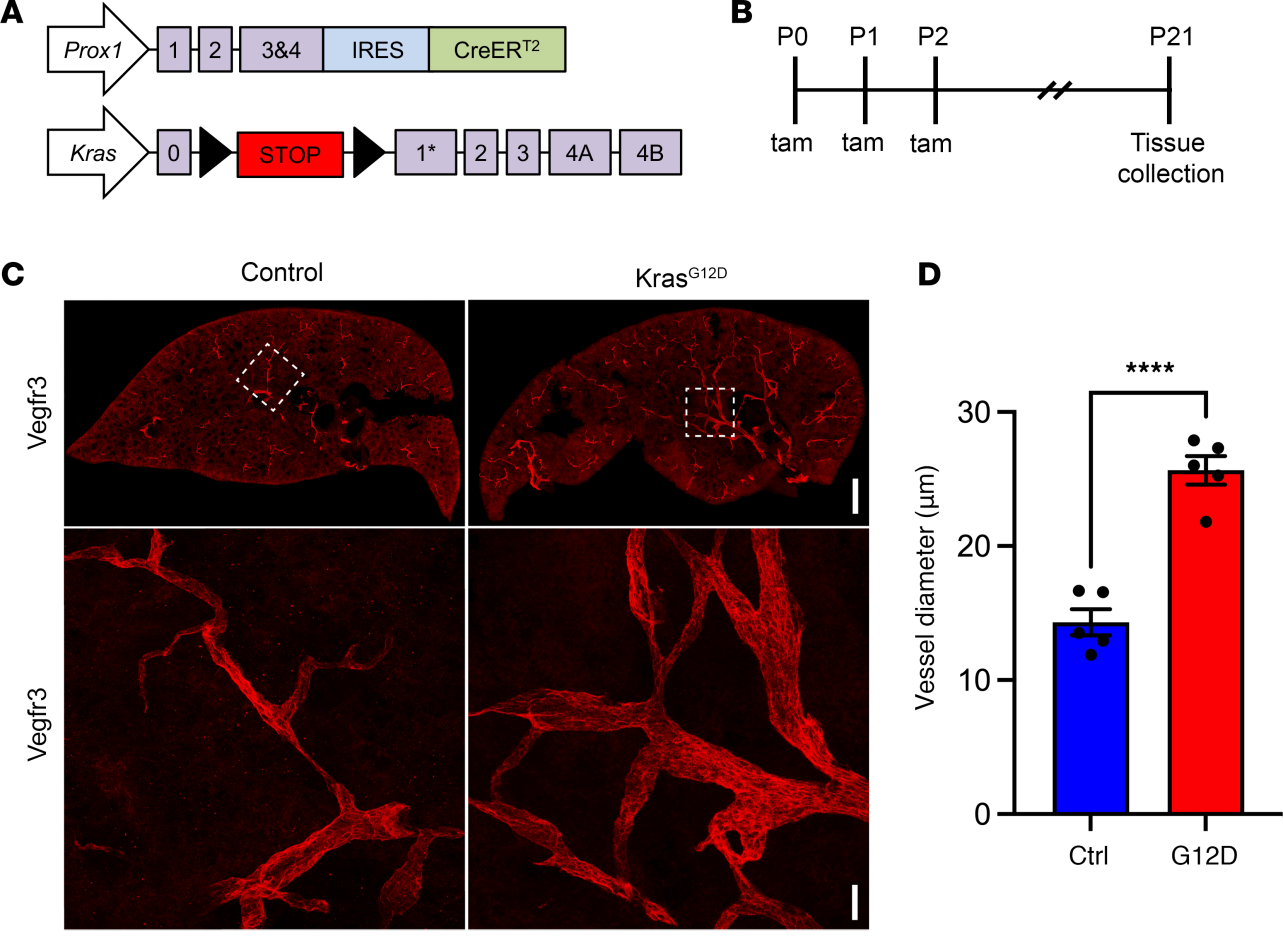
Pulmonary lymphatic vessels are enlarged in Kras^G12D^ mice. (**A**) Schematics of the *Prox1-CreER^T2^* and *Kras^LSL-G12D^* alleles. (**B**) Schematic showing when mice received tamoxifen (50 μg; p.o.). Tissues were collected when mice were 21 days old. (**C**) Low- and high-magnification views of Vegfr3-positive lymphatic vessels in 100-μm-thick lung sections from control and Kras^G12D^ mice. The high-magnification images are of the boxed regions. (**D**) The diameter of lymphatic vessels was significantly greater in Kras^G12D^ mice (25.65 ± 1.070 μm; *n* = 5) than in control (Ctrl) mice (14.31 ± 0.9752 μm; *n* = 5). Data are presented as mean ± SEM. ****P < 0.0001 by 2-tailed, unpaired Student’s *t* test. Scale bars: 500 μm (low magnification) and 50 μm (high magnification).

We previously reported that Kras^G12D^ impairs the development of lymphatic vessels in the skin of mice (17, 20). To determine whether Kras^G12D^ exerts similar effects on lymphatic vessels in other tissues, we analyzed pulmonary lymphatic vessels in *Prox1-CreER^T2^;Kras^wt/wt^* (control) and *Prox1-CreER^T2^;Kras^wt/LSL-G12D^* (Kras^G12D^) mice (Figure 1A). *Prox1-CreER^T2^* mice express a tamoxifen-inducible form of Cre recombinase in LECs, and *Kras^LSL-G12D^* mice express an active form of Kras (KRAS.pG12D) from the endogenous *Kras* locus following Cre-mediated recombination. Newborn mice were fed tamoxifen from postnatal day 0 (P0) to P2 to induce Cre-mediated recombination in *Prox1*-positive cells (Figure 1B). Following tamoxifen administration, lungs from 3-week-old control and Kras^G12D^ mice were collected, sectioned, and immunostained for Vegfr3 (Figure 1C). Consistent with what we previously reported for the skin (17, 20), we found that the diameter of pulmonary lymphatic vessels was significantly greater in Kras^G12D^ mice than in control mice (Figure 1D).

**Figure 2:**
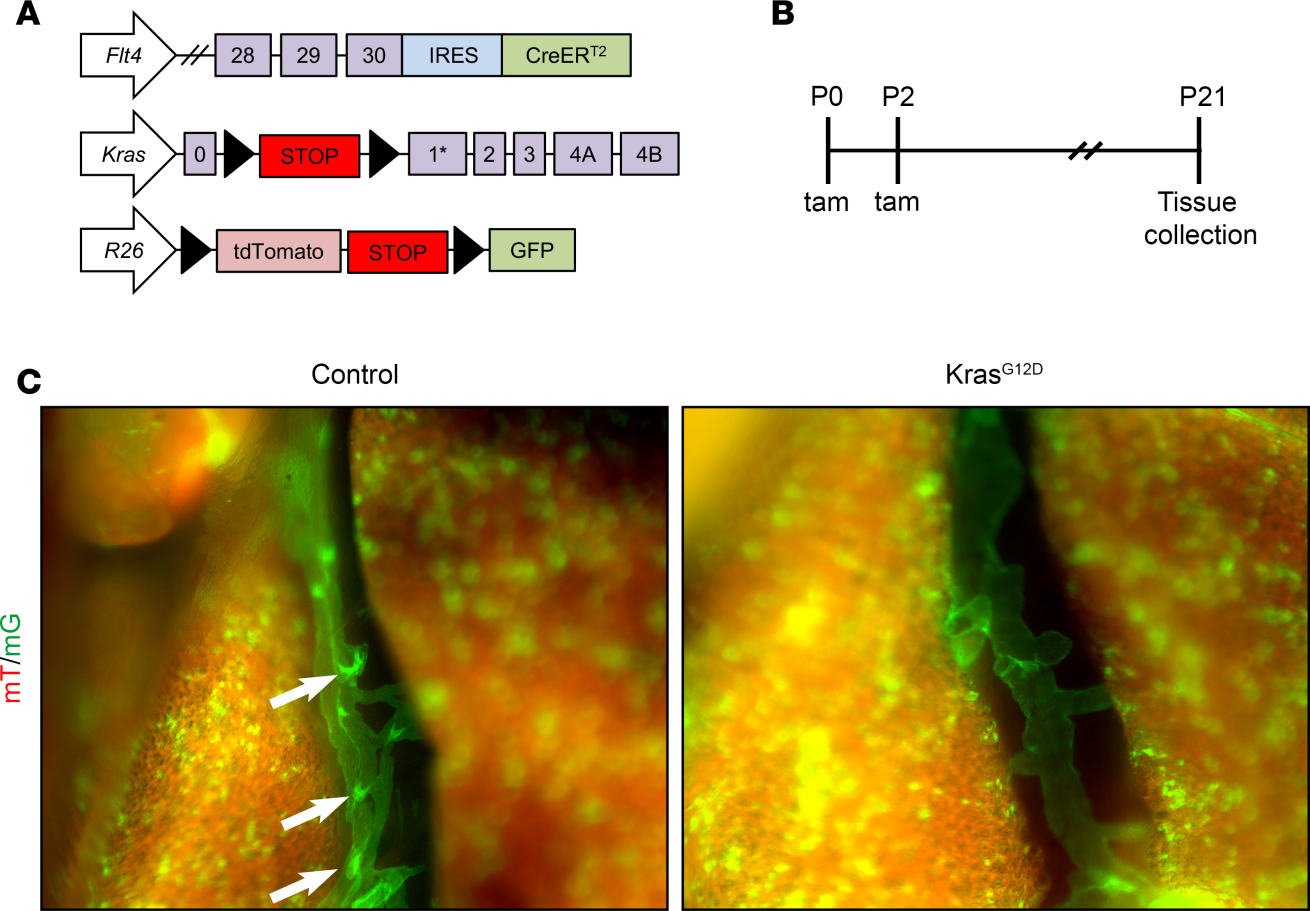
Kras^G12D^ impairs lymphatic valve development. (**A**) Schematics of the *Flt4-CreER^T2^*, *Rosa26^mT/mG^* (*R26^mT/mG^*), and *Kras^LSL-G12D^* alleles. (**B**) Schematic showing when mice received tamoxifen (100 μg; s.c.). Tissues were collected when mice were 21 days old. (**C**) GFP-positive interlobular lymphatic vessels in control and Kras^G12D^ mice. Lymphatic valves (arrows) were readily observed in control mice (*n* = 4 mice), but not in Kras^G12D^ mice (*n* = 3 mice).

To further characterize the effect of Kras^G12D^ on pulmonary lymphatic vessels, we used the *Vegfr3-CreER^T2^* strain to drive Kras^G12D^ expression in LECs in newborn mice (Figure 2A). We included the *Rosa26^mT/mG^* (hereafter, *mT/mG*) reporter strain in our experiment to label LECs that had undergone Cre-mediated recombination with green fluorescent protein (GFP). Newborn mice received tamoxifen on P0 and P2, and lungs were harvested 3 weeks later for analysis (Figure 2B). We readily observed lymphatic valves in interlobular lymphatic vessels in control mice, but not in Kras^G12D^ mice (Figure 2C). These results suggest that Kras^G12D^ impairs the formation of lymphatic valves in pulmonary lymphatic vessels.

### scRNA-Seq reveals 6 LEC subtypes in the lungs of control mice.

**Figure 3:**
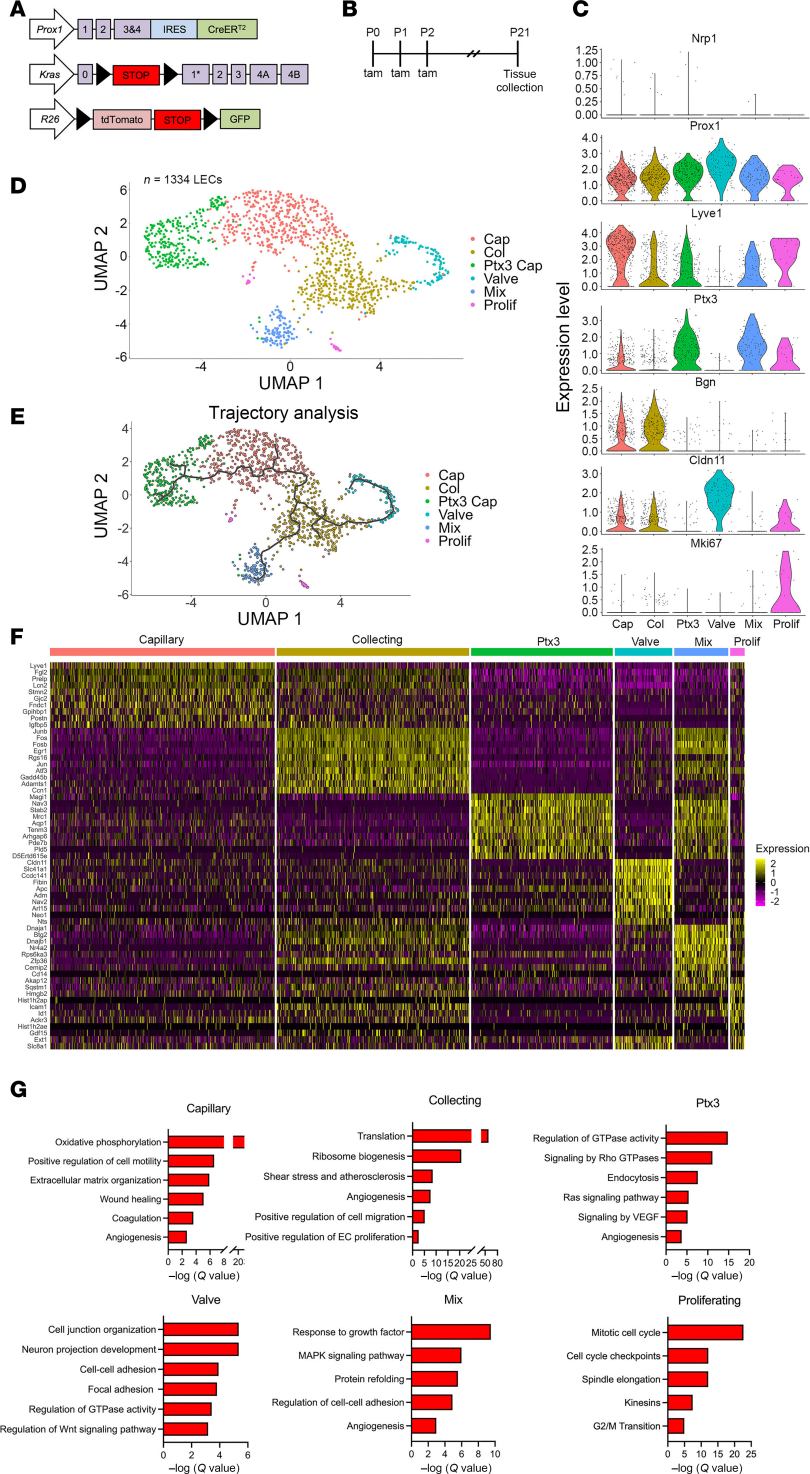
The cellular and transcriptional profile of pulmonary LECs in control mice revealed by scRNA-Seq. (**A**) Schematics of the *Prox1-CreER^T2^*, *R26^mT/mG^*, and *Kras^LSL-G12D^* alleles. (**B**) Schematic showing when mice received tamoxifen (50 μg; p.o.). Tissues were collected when mice were 21 days old. (**C**) Violin plots showing the expression of select blood endothelial cell and LEC markers and LEC subtype markers. (**D**) UMAP showing the clustering of LECs (*n* = 1,334 cells) from control mice. We identified 6 unique clusters of LECs in the lungs of control mice. (**E**) Trajectory analysis of LECs from control mice using Monocle 3. (**F**) Heatmap showing the top 10 most differentially expressed genes for each LEC subtype. (**G**) GO terms analysis of genes enriched in each LEC subtype.

Next, we used scRNA-Seq to investigate the effects of Kras^G12D^ on the cellular and transcriptional landscape of pulmonary lymphatic vessels. Like before, we included the *mT/mG* reporter strain in our breeding scheme to label Prox1-positive cells that had undergone Cre-mediated recombination with GFP (Figure 3A). Newborn mice were fed tamoxifen from P0 to P2 to induce Cre-mediated recombination in *Prox1*-expressing cells (Figure 3B). Following tamoxifen administration, lungs from 3-week-old control (*n* = 5 mice) and Kras^G12D^ mice (*n* = 4 mice) were dissociated into single-cell suspensions, and live GFP-positive cells were sorted by FACS. The cells were then analyzed by scRNA-Seq using 10× Genomics 3′ chemistry. We first analyzed the cellular identities and transcriptional profiles of control LECs. After quality control (QC) and filtering, we obtained data for 1,334 control LECs. These cells were used to run Seurat’s graph-based clustering method. Cell clustering data were visualized by Uniform Manifold Approximation and Projection (UMAP). We observed 6 clusters and all clusters expressed endothelial cell markers (*Cdh5*, *Kdr*, and *Pecam1*; Figure 3D and Supplemental Figure 1A; supplemental material available online with this article; https://doi.org/10.1172/jci.insight.185181DS1). We assessed the purity of the LEC population by confirming the expression of LEC-specific markers (*Prox1*, *Nrp2*, and *Pdpn*; Figure 3C and Supplemental Figure 1A) and the absence of blood endothelial cell–specific markers (*Vwf* and *Nrp1*; Figure 3C and Supplemental Figure 1A). Additionally, we ruled out significant contamination by immune cells by confirming the absence of immune cell markers (*Ptprc*, *Itgam*, *Adgre1*, *Cd19*, and *Cd3e*) in the dataset (Supplemental Figure 2). The clusters were then annotated based on the expression of previously reported markers of LEC subtypes (Figure 3C and Supplemental Figure 1) (21, 22). We identified 6 subtypes of lymphatic endothelium in control mice, including capillary (*Lyve1^hi^*), collecting (*Bgn^hi^* and *Foxp2^hi^*), valve (*Cldn11^hi^*), proliferating (*Mki67^hi^*), and Ptx3 LECs (*Ptx3^hi^*) (Figure 3, C and D). Additionally, we identified a mixed population of LECs (Figure 3, C and D) that expressed markers common to Ptx3 and collecting LECs (Figure 3C and Supplemental Figure 1B).

We then characterized the relationship between the different LEC subtypes by modeling their transcriptional trajectories. Our trajectory analysis revealed a continuous trajectory that extended from the Ptx3 cluster, through the capillary and collecting clusters, before bifurcating into the mixed and valve clusters (Figure 3E). We noticed additional branches with leaflets in every cluster, except the mixed and valve clusters, where the trajectories terminated in single leaflets (Figure 3E). To determine the transcriptional profile of the different LEC subtypes, we performed differential gene expression (DGE) analysis. Our DGE analysis revealed that each subtype of lymphatic endothelium possessed a distinct transcriptional profile (Figure 3F, Supplemental Figure 1B, and Supplemental Dataset 1). In addition to finding unique markers of the different LEC subtypes, we identified genes previously reported (21, 22) to be specific to capillary (*Lyve1*, *Fgl2*, *Stmn2*, *Fndc1*, *Piezo2*, and *Adgrg3*), Ptx3 (*Ptx3*, *Mrc1*, *Aqp1*, and *Itih5*), collecting (*Apoe*, *Bgn*, *Procr*, and *Foxp2*), valve (*Cldn11*, *Slc41a1*, *Fibin*, *Arl15*, *Adm*, *Neo1*, and *Net1*)*,* and proliferating (*Mki67* and *Ccnb2*) LECs (Figure 3F, Supplemental Figure 1B, and Supplemental Dataset 1), suggesting that the transcriptional profile of LEC subtypes is similar across tissues.

To identify biological processes that may contribute to the development and maintenance of the different LEC subtypes, we performed gene ontology (GO) analysis (Supplemental Dataset 2). Our analysis showed that genes associated with oxidative phosphorylation, cell motility, and extracellular matrix organization were enriched in the capillary cluster (Figure 3G). In the collecting cluster, translation, ribosome biogenesis, and cell migration were among the upregulated GO terms (Figure 3G). Some of the GO terms enriched in the valve cluster included cell junction organization, cell-cell adhesion, and focal adhesion (Figure 3G). As expected, pathways related to cell cycle and proliferation were enriched in the proliferating cluster (Figure 3G). Interestingly, the Ptx3 and mixed clusters displayed terms related to growth factor signaling and Ras or MAPK signaling (Figure 3G), raising the possibility that these 2 LEC subtypes may be more susceptible to perturbations in Ras or MAPK signaling.

### Developmental atlas of lung LECs.

**Figure 4:**
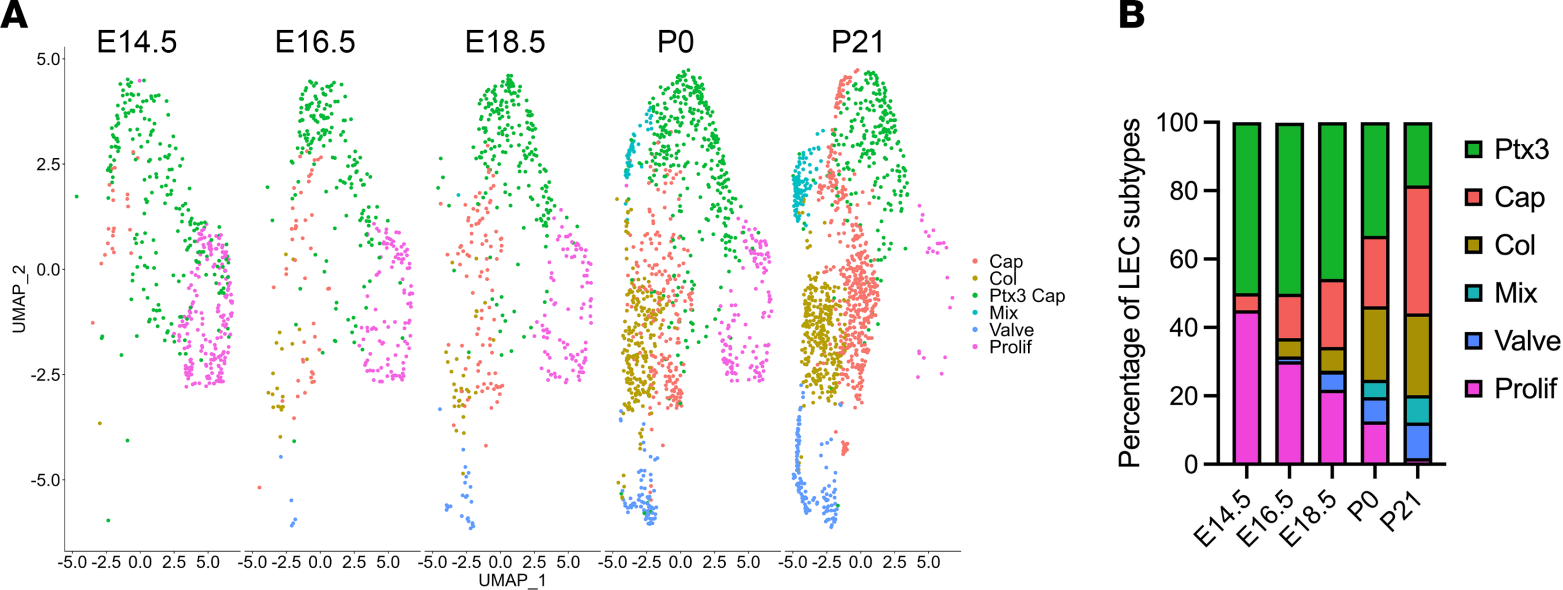
Temporal mapping of the pulmonary LEC landscape. (**A**) UMAPs showing the pulmonary LEC subtypes present at E14.5, E16.5, E18.5, P0, and P21. (**B**) Graph showing the percentage of the LEC subtypes present at E14.5, E16.5, E18.5, P0, and P21.

During development, an immature network of capillary-like lymphatic vessels emerges, with a portion of these vessels eventually maturing into collecting vessels with valves (25, 26). The stepwise specification of LEC subtypes during development has not been extensively characterized by scRNA-Seq. To determine when the different LEC subtypes arise during development, we integrated our control scRNA-Seq dataset (P21) with scRNA-Seq datasets for lung LECs that span 4 developmental time points (E14.5, E16.5, E18.5, and P0) (27). The integrated data were then clustered, visualized by UMAP, and annotated based on the expression of subtype-specific markers. We observed 6 clusters in the integrated dataset, corresponding to Ptx3, capillary, collecting, mixed, valve, and proliferating LECs. We found that Ptx3 and proliferating LECs were the predominant LEC subtypes at E14.5. However, the proportion of Ptx3 and proliferating LECs decreased as the lymphatic network developed from E14.5 to P21 (Figure 4, A and B). Conversely, the proportion of capillary, collecting, mixed, and valve LECs increased as the lymphatic network matured from E16.5 to P21 (Figure 4, A and B).

### LECs in Kras^G12D^ mice have an altered transcriptional profile.

Next, we analyzed LECs isolated from the lungs of 3-week-old Kras^G12D^ mice. After performing QC and filtering, we obtained data for 2,723 Kras^G12D^ LECs. Interestingly, cell clustering and visualization by UMAP revealed only 5 clusters of LECs (Supplemental Figure 3). Based on the expression of subtype-specific markers, we identified clusters of Ptx3, capillary, mixed, collecting, and proliferating LECs. Notably, a cluster of valve LECs was absent for Kras^G12D^ mice (Supplemental Figure 3). We next performed trajectory analysis to determine the transcriptional relationship of Kras^G12D^ LECs within the different clusters. Our analysis revealed continuous trajectories that extended from the Ptx3 cluster, through the capillary and collecting clusters, and terminated in the mixed cluster (Supplemental Figure 3). Importantly, we noticed an absence of a trajectory bifurcation in the collecting cluster (Supplemental Figure 3). We also noted the presence of looped trajectories in the Ptx3 and capillary clusters, which may suggest transcriptional heterogeneity within the clusters (Supplemental Figure 3). We then performed DGE analysis to characterize the transcriptional landscape of the different subtypes of Kras^G12D^ LECs. We found that many of the top markers of the control LEC subtypes did not show up among the top differentially expressed genes of the Kras^G12D^ LEC subtypes (Supplemental Figure 3), suggesting that hyperactive KRAS signaling alters the transcriptional profile of LECs.

### Kras^G12D^ alters the cellular landscape of lymphatic vessels.

**Figure 5:**
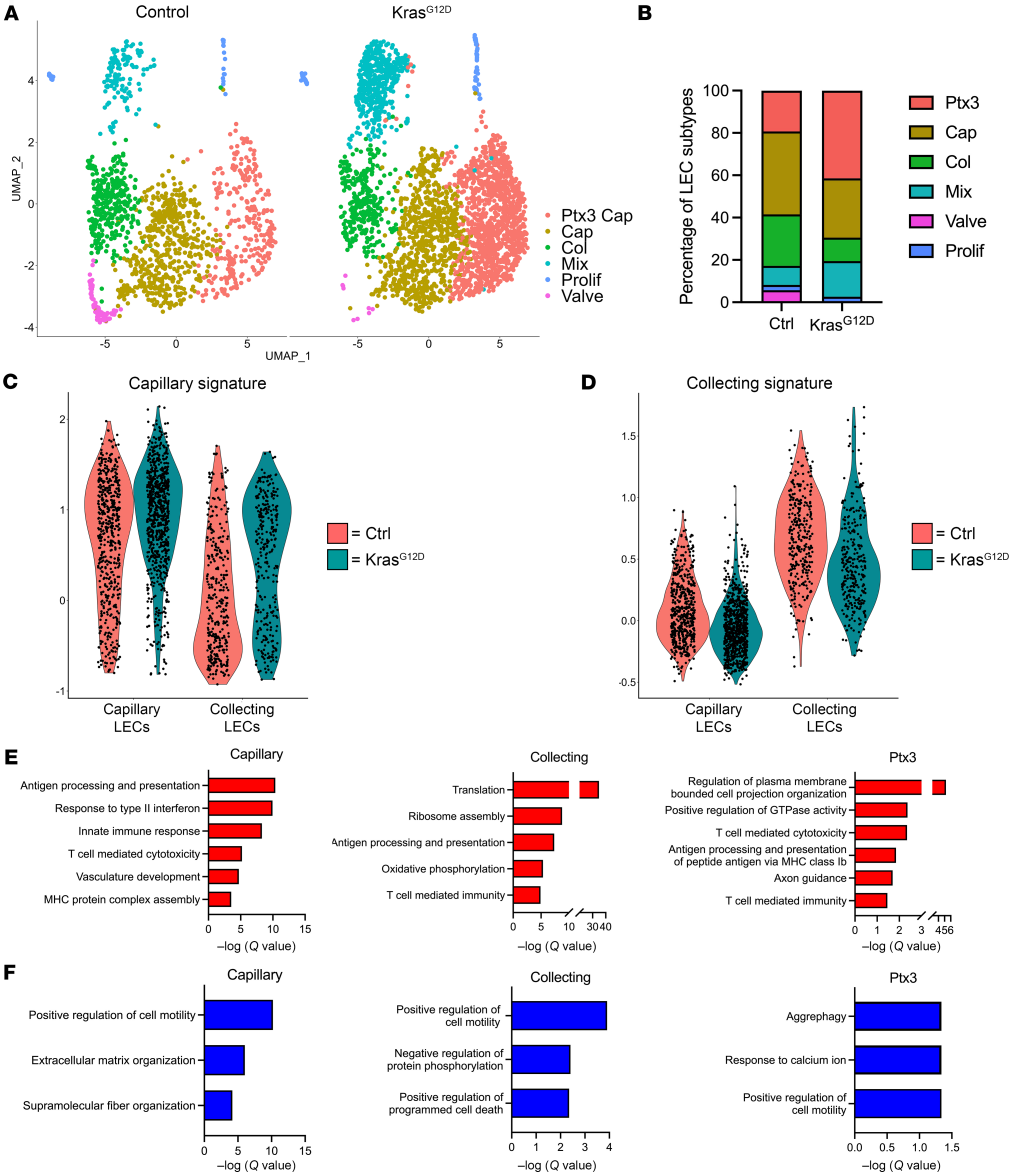
Kras^G12D^ induces changes in the cellular and transcriptional landscape of lymphatic vessels. (**A**) UMAPs for pulmonary LECs from control and Kras^G12D^ mice. (**B**) Graph showing the percentage of the LEC subtypes in the lungs of control and Kras^G12D^ mice. (**C**) Violin plots showing average gene expression module scores for capillary signature genes. (**D**) Violin plots showing average gene expression module scores for collecting vessel signature genes. (**E**) GO terms associated with genes upregulated by Kras^G12D^. (**F**) GO terms associated with genes downregulated by Kras^G12D^.

To further investigate the cellular and molecular changes caused by Kras^G12D^, we integrated the control and Kras^G12D^ datasets using Seurat’s canonical correlation analysis–based integration method. After integration, QC, and filtering, we obtained data for 1,341 control and 2,727 Kras^G12D^ LECs (Figure 5A). Marker-based annotation revealed clusters of Ptx3, capillary, collecting, mixed, proliferating, and valve LECs (Figure 5A). We found that the proportion of Ptx3 and mixed LECs was greater in Kras^G12D^ mice than in control mice (Figure 5B). Conversely, the proportions of valve, collecting, and capillary LECs were lower in Kras^G12D^ mice compared with control mice (Figure 5B). Interestingly, the cellular landscape of lymphatic vessels in Kras^G12D^ mice was similar to that in control embryos, suggesting that Kras^G12D^ impairs the maturation of lymphatic vessels.

We previously reported that Kras^G12D^ mice have fewer lymphatics exhibiting characteristics of collecting vessels than control mice (17). To examine this at the single-cell level, we scored the average gene expression levels of capillary and collecting LEC signature genes derived from control mice. Our analysis revealed that collecting LECs from Kras^G12D^ mice have a higher capillary LEC signature score and lower collecting LEC signature score than collecting LECs from control mice (Figure 5, C and D). These data suggest that collecting LECs in Kras^G12D^ mice display an immature capillary-like transcriptional profile (Figure 5, C and D).

We further characterized the transcriptional differences between control and Kras^G12D^ LECs by performing DGE analysis (Supplemental Dataset 3). To identify biological processes potentially impacted by Kras^G12D^, we examined the enrichment of GO terms in the differentially expressed genes (Supplemental Dataset 4). Due to the presence of very few differentially expressed genes in the mixed and proliferating clusters and very low number of cells in the valves of mutant mice, GO analysis was not performed on these LEC subtypes. We found that genes associated with various immune-related processes such as response to type II IFN (*Gbp2*, *Gbp6*, *Gbp7*, and *Gbp9*), innate immune response (*C3*, *Mrc1*, *Ccl21a*, and *Ptx3*), and antigen processing and presentation (*B2m*, *H2-D1*, *H2-K1*, and *H2-Q7*) were upregulated in Kras^G12D^ LECs (Figure 5E). Interestingly, genes involved in positive regulation of cell motility (*Cdh13*, *Col18a1*, *Hspb1*, and *Gstp1*) were downregulated in Kras^G12D^ LEC subtypes compared with control LECs (Figure 5F).

### Kras^G12D^ exerts a more profound effect on immature than mature lymphatic vessels.

**Figure 6:**
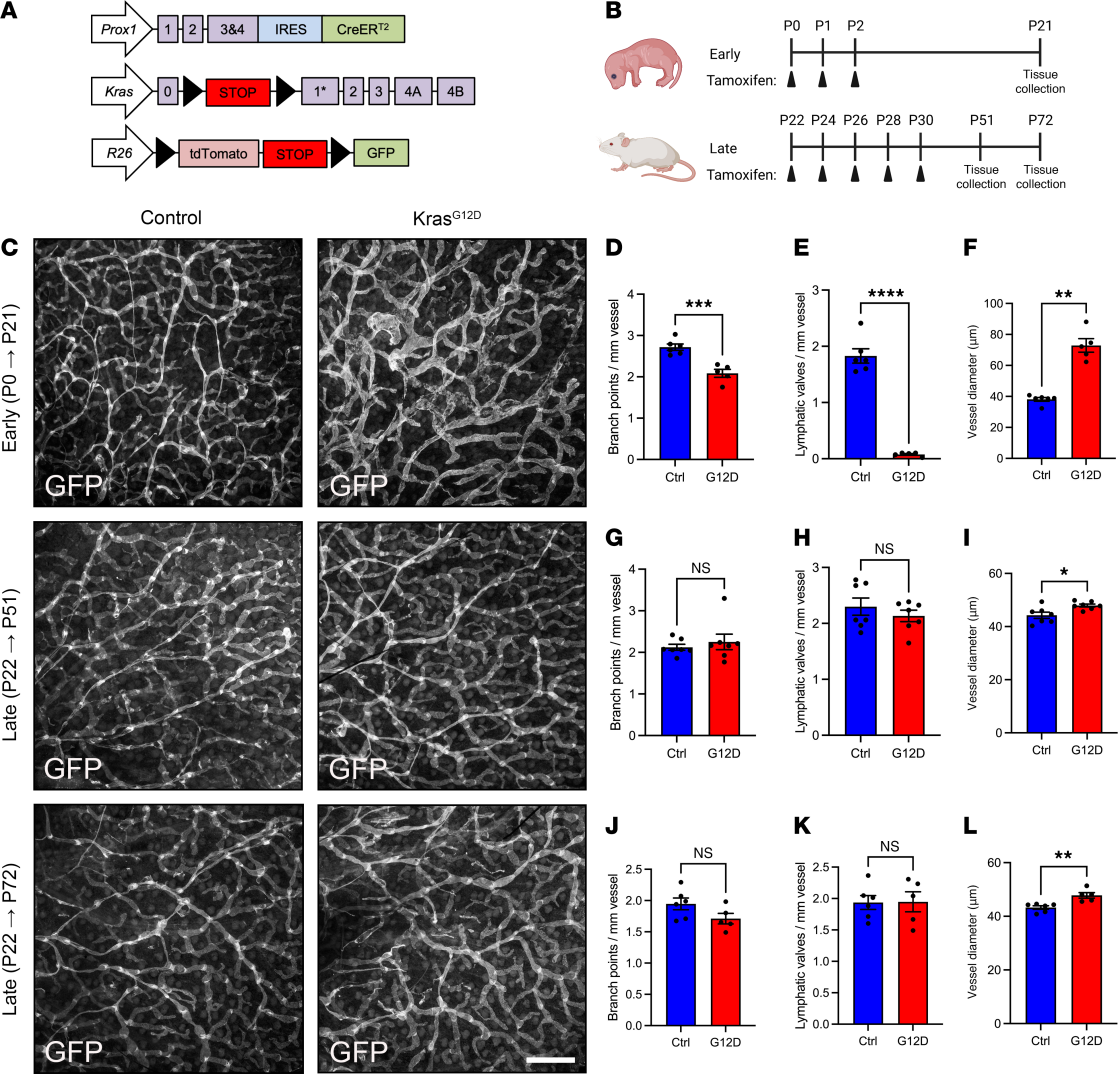
Temporal sensitivity of lymphatic vessels to Kras^G12D^. (**A**) Schematics of the *Prox1-CreER^T2^*, *R26^mT/mG^*, and *Kras^LSL-G12D^* alleles. (**B**) Schematic showing when mice received tamoxifen (early induction = 50 μg, p.o.; late induction = 2 mg, i.p.). (**C**) Representative images of ear skin whole mounts stained with an anti-GFP antibody. (**D**–**F**) Results for early tamoxifen treatment. (**D**) Branch points per millimeter vessel for control (2.718 ± 0.07512; *n* = 6) and Kras^G12D^ (2.086 ± 0.09636; *n* = 5) mice. (**E**) Valves per millimeter vessel for control (1.828 ± 0.1274; *n* = 6) and Kras^G12D^ (0.0760 ± 0.01503; *n* = 5) mice. (**F**) Vessel diameters for control (38.12 ± 1.212 μm; *n* = 6) and Kras^G12D^ (72.89 ± 4.360 μm; *n* = 5) mice. (**G**–**I**) Results for late tamoxifen treatment and early collection. (**G**) Branch points per millimeter vessel for control (2.122 ± 0.07161; *n* = 7) and Kras^G12D^ (2.252 ± 0.1862; *n* = 7) mice. (**H**) Valves per millimeter vessel for control (2.299 ± 0.1534; *n* = 7) and Kras^G12D^ (2.133 ± 0.1016 *n* = 7) mice. (**I**) Vessel diameters for control (44.25 ± 1.164 μm) and Kras^G12D^ (47.92 ± 0.5579 μm) mice. (**J**–**L**) Results for late tamoxifen treatment and late collection. (**J**) Branch points per millimeter vessel for control (1.947 ± 0.09457; *n* = 6) and Kras^G12D^ (1.711 ± 0.08402; *n* = 5) mice. (**K**) Valves per millimeter vessel for control (1.937 ± 0.1105; *n* = 6) and Kras^G12D^ (1.948 ± 0.1595; *n* = 5) mice. (**L**) Vessel diameters for control (43.26 ± 0.6543 μm; *n* = 6) and Kras^G12D^ (47.81 ± 1.036 μm; *n* = 5) mice. Data are presented as mean ± SEM. NS, not significant, *P < 0.05, **P < 0.01, ***P < 0.001, ****P < 0.0001 by 2-tailed, unpaired Student’s *t* test (**D**, **E**, **H**, **I**, **J**, **K**, and **L**) or Mann-Whitney test (**F** and **G**). Scale bar: 500 μm.

Our scRNA-Seq data suggest that Kras^G12D^ impairs the maturation of lymphatic vessels. That finding prompted us to examine the effect of Kras^G12D^ on immature and mature lymphatic vessels. We used our previously described model (Figure 3A) to express Kras^G12D^ and GFP in *Prox1*-expressing cells. To test the effect of Kras^G12D^ on immature lymphatic vessels, we fed newborn mice tamoxifen from P0 to P2 (Figure 6, A and B). Three weeks after feeding the mice tamoxifen, we stained their ears with an anti-GFP antibody (Figure 6C). We focused our analysis on ears because the lymphatic network in the ear develops postnatally and is mature (i.e., possesses collecting lymphatic vessels that contain valves and are surrounded by lymphatic muscle cells) by P21 (17). We found that Kras^G12D^ mice had significantly fewer lymphatic branch points and valves than control mice (Figure 6, D and E). Additionally, the diameter of lymphatic vessels was significantly greater in Kras^G12D^ mice than in control mice (Figure 6F). However, the number of Prox1-positive nuclei per millimeter of lymphatic vessel was not significantly different between control and Kras^G12D^ mice (Supplemental Figure 4, A–C). To characterize the effect of Kras^G12D^ on mature lymphatic vessels, we injected mice with tamoxifen from P22 to P30. We collected ears from mice 3 or 6 weeks after they received their last tamoxifen injection and stained them with an anti-GFP antibody. We found that the number of lymphatic branch points and valves was not significantly different between control and Kras^G12D^ mice (Figure 6, G, H, J, and K). Additionally, the number of Prox1-positive nuclei per millimeter of lymphatic vessel was not significantly different between control and Kras^G12D^ mice (Supplemental Figure 4, D–F). However, the diameter of lymphatic vessels was slightly increased in Kras^G12D^ mice (Figure 6, I and L). Together, these data suggest that immature lymphatic vessels in young mice are more sensitive to the pathologic effects of Kras^G12D^ than mature lymphatic vessels in older mice.

## Discussion

Somatic activating mutations in *KRAS* have previously been shown to cause CLAs. However, the effect of *KRAS* mutations on LEC heterogeneity has not been explored. In the present study, we use scRNA-Seq to characterize the cellular and transcriptional landscape of normal and KRAS^G12D^-malformed lymphatic vessels. We show that KRAS^G12D^ expression in LECs during postnatal development leads to an increase in Ptx3 LECs and a decrease in collecting and valve LECs, resulting in a cellular profile similar to that of normal embryos. We also show that growing lymphatic vessels demonstrate a greater sensitivity to the pathologic effects of Kras^G12D^ than mature lymphatic vessels. Together, these results suggest that hyperactive KRAS signaling in LECs impairs the remodeling and maturation of lymphatic vessels.

scRNA-Seq has become a valuable tool for examining the diversity of cell populations and has greatly enhanced our ability to identify subtypes of LECs (21–24). However, scRNA-Seq has not been widely used to determine when the different LEC subtypes are specified during development. We found that proliferating and Ptx3 LECs are the predominant LEC subtypes during early development. Later in development, we saw the emergence of capillary, collecting, and then valve LECs. Trajectory analysis revealed a linear path from Ptx3 to capillary, collecting, and valve LECs. These findings expand the current model for the development of the lymphatic network and raise the possibility that Ptx3 LECs give rise to capillary LECs, which further differentiate into collecting and then valve LECs. In the future, lineage tracing experiments with *Ptx3-Cre^ERT2^* mice could reveal the developmental relationship between Ptx3 LECs and other LEC subtypes.

We previously reported that Kras^G12D^ suppresses the recruitment of lymphatic muscle cells to lymphatic vessels, the specification of collecting vessels (e.g., downregulation of Lyve1), and the formation of lymphatic valves (17, 20). Those observations led us to propose that Kras^G12D^ hinders the maturation of lymphatic vessels. In agreement with our previous work, we show here by scRNA-Seq that Kras^G12D^ mice have fewer collecting and valve LECs than control mice and that collecting LECs in Kras^G12D^ mice have a higher capillary gene signature than collecting LECs in control mice. We also show that the proportion of Ptx3 LECs decreases during normal development as the lymphatic network matures, but remains high in Kras^G12D^ mice. These new results extend our previous observations and further suggest that Kras^G12D^ suppresses lymphatic remodeling and maturation.

We have found that immature lymphatic vessels in young mice are more sensitive to the pathologic effects of Kras^G12D^ than mature lymphatic vessels in older mice. Similar observations have recently been reported for mice that express an active form of Nras (Nras^Q61R^) in their LECs, where Nras^Q61R^ induces LMs in embryos but not in newborns (28). This temporal sensitivity to genetic mutations could explain why CLAs are typically diagnosed in children and young adults. Although there appears to be a developmental window in which lymphatic vessels are highly sensitive to Kras^G12D^ and Nras^Q61R^, such a window has not been reported for Pik3ca^H1047R^. Somatic activating mutations in PIK3CA can cause CLAs/LMs, and we and others have found that Pik3ca^H1047R^ can induce LMs from stable lymphatic vessels (12, 21, 29). The differences between Kras^G12D^, Nras^Q61R^, and Pik3ca^H1047R^ mice could be related to the design of the different mouse lines. Following Cre-mediated recombination, the Kras^G12D^ and Nras^Q61R^ mutations are expressed from their endogenous loci, whereas the Pik3ca^H1047R^ mutation is expressed from the Rosa26 locus. Alternatively, inherent differences between the genes or in the strength of the mutations could explain differences in the temporal sensitivity of lymphatic vessels to the mutations.

There is emerging evidence that immune cells function in the pathogenesis of CLAs/LMs (21). It was recently reported that Pik3ca^H1047R^ drives the expansion of the Ptx3 LEC population, which in turn recruits VEGF-C–expressing macrophages that stimulate lymphangiogenesis (21). We found that the population of Ptx3 LECs was increased in Kras^G12D^ mice and that many of the differentially expressed genes between control and Kras^G12D^ mice were involved in various immune processes (e.g., innate immune response, MHC protein complex 1 assembly, antigen processing and presentation, and T cell–mediated immunity). In the future, experiments with inhibitors that block the recruitment or activity of different immune cell populations could reveal whether immune cells drive the progression of Kras^G12D^-induced LMs, as they do Pik3ca^H1047R^-induced LMs.

Although activating mutations in KRAS had been identified in patients with CLA, the precise mechanisms by which the mutations cause CLAs are poorly understood. Our findings indicate that KRAS mutations keep lymphatic vessels in an immature state, leading to an imbalance in LEC subtypes. Together, our results shed light on the mechanisms regulating LEC heterogeneity and how KRAS mutations cause CLAs.

### Study limitations.

The study of Ptx3 LECs has faced significant challenges due to the difficulty in identifying these cells. The lack of tools, such as antibodies and reporter mice, has limited the scope of research on Ptx3 LECs. One of the limitations of our study is that we do not resolve the spatial location of Ptx3 and mixed LECs in the lungs of mice. In the future, new reporter and CreER^T2^ lines could help clarify the anatomic location of these cells.

Interestingly, several stress-induced genes (e.g., *Atf3*, *Fos*, *Fosb*, *Jun*, *Junb*, and *Socs3*) are expressed by collecting LECs in our data set. These stress-induced genes are also highly expressed by collecting LECs in a publicly available scRNA-Seq data set of mesenteric lymphatics (22). The expression of these stress-induced genes could be biologically relevant. Collecting lymphatic vessels contract to pump lymph against a pressure gradient and are exposed to shear forces ranging from 0 to 12 dynes/cm^2^ (30). Exposure to these stresses could promote the expression of these stress-responsive genes by collecting LECs. However, the expression of some stress-induced genes has been proposed to be caused by tissue dissociation protocols (31). Presently, we cannot rule out the possibility that the expression of some of these genes might be artifacts caused by tissue dissociation.

## Methods

*Sex as a biological variable*. Our study examined male and female animals, and similar findings are reported for both sexes.

*Mouse strains and genotyping*. Mice were maintained in ventilated microisolator cages and were fed a standard diet. Mice were provided igloos and nestlets as enrichment items. The *LSL-Kras^G12D^*, *Prox1-CreER^T2^*, *Flt4-CreER^T2^*, and *mT/mG* strains were described previously (32–35). Established protocols were followed to genotype the mice (17).

### Tamoxifen

#### Newborn mice.

We dissolved tamoxifen (25 mg; MilliporeSigma, T5648) in a mixture of ethanol (100 μL; MilliporeSigma, E7023) and peanut oil (900 μL; MilliporeSigma, P2144). To induce Cre-mediated recombination, we fed newborn mice 2 μL of tamoxifen on P0, P1, and P2 using a P20 pipette. Alternatively, tamoxifen was dissolved in sunflower oil and ethanol for a final stock of 20 mg/mL with 5% ethanol. Mice received a subcutaneous injection (100 μg) in the nape of the neck on P0 and P2.

#### Young adult mice.

We dissolved 20 mg tamoxifen in a mixture of 100 μL ethanol and 900 μL sunflower oil (MilliporeSigma, W530285). To induce Cre-mediated recombination, we injected mice (i.p.) with 100 μL of tamoxifen on P22, P24, P26, P28, and P30.

### Single-cell isolation, sorting, and sequencing

Lungs from male and female mice were pooled together to isolate LECs from groups of control and Kras^G12D^ mice. The lungs were perfused with heparin/DPBS pH 7.2 to remove red blood cells. The lobes of the lung were isolated and cut into pieces with a scalpel before placing them into C-tubes (Miltenyi Biotec, 130-093-237) containing 5 mL of a lung dissociation cocktail (Miltenyi Biotec, 130-095-927). The C-tubes containing the tissue/enzyme mix were then placed into the sleeve of a gentleMACS Dissociator (Miltenyi Biotec, 130-093-235), which is a benchtop instrument used to dissociate tissues into single-cell suspensions. We ran the preset m_lung_01 program for 8 seconds to dissociate the lung tissue. The C-tubes were then placed in a 37°C incubator under continuous agitation for 45 minutes. Following incubation, the C-tubes containing the tissue/enzyme mix were placed into the gentleMACS Dissociator, and the preset m_lung_02 program was run for 38 seconds to dissociate the cells. The C-tubes were then centrifuged at 300*g* to collect the sample at the bottom of the tube. The sample was resuspended with 1× Buffer S and applied to a prewet 100-μm cell strainer in a 50 mL conical tube. This process was repeated with a 70 μm and 40 μm cell strainer to ensure that all cells were in a single-cell suspension. The cell suspension was transferred to a 15 mL conical tube and centrifuged at 300*g* to collect a pellet. The pellet was resuspended in 3% FBS in 1× DPBS for subsequent FACS analysis. The FACSAria II (BD BioSciences) was used for sorting LECs. Cells were sorted at a temperature of 4°C with a 100 μm nozzle at a pressure of 100 psi. Events were first gated on FSC-A versus SSC-A. Then, events were gated on FSC-A versus FSC-W and SSC-W versus SSC-A for gating singles cells. Dead cells were then labeled with DRAQ7 far-red viability dye (Novus Biologicals, NBP2-81126) and events were gated for live cell populations. Finally, cells were gated on GFP-A versus tdTomato-A to sort all GFP-positive LECs. The sorted LECs were then submitted for sequencing. The cells were sequenced by the University of Texas Southwestern Medical Center genomics core. The Chromium Next GEM Single Cell Reagent Kits v3.1 Dual Index (10× Genomics, PN-1000269) was used for library preparation according to the manufacturer’s protocol. Single Cell 3′ v3 Chemistry was used for library preparation. The libraries were sequenced using the NovaSeq 6000 sequencing system (Illumina). Demultiplexing was performed using the bcl2fastq v2.20.0 software (Illumina) and Cell Ranger v7.0.0 (10× Genomics) was used for barcode processing, gene counting, and aggregation.

### scRNA-Seq data processing

The Seurat package (v4.4.0) (36) was used to analyze the scRNA-Seq data in R-studio (R v4.3.1). Briefly, raw data from the 10× Genomics Cell Ranger pipeline was loaded into Seurat to generate a UMI count matrix. The count matrix was utilized to create a Seurat object to use in subsequent analysis. While creating the Seurat object, we performed an initial filtration step to remove very-low-quality cells and speed up downstream computational processing. We set the min.cells and min.features arguments to include features that are expressed in at least 3 cells and cells expressing at least 200 genes. After creating the Seurat object, we visualized the QC metrics and performed a second filtration step. Cells that had more than 500 unique features and a percentage mitochondrial count less than 5% were retained. The default parameters were used for control and Kras^G12D^ datasets to normalize the counts, find variable features, scale the data, and perform PCA dimensionality reduction using the NormalizeData(), FindVariableFeatures(), ScaleData(), and RunPCA() functions of Seurat, respectively. To cluster control and Kras^G12D^ datasets, we used Seurat’s FindNeighbors() and FindClusters() functions using 19 PCs and a resolution of 0.3. Dimensionality reduction was performed using the UMAP method implemented in Seurat’s RunUMAP() function with the number of PCs set to 19. The UMAPs were visualized using Seurat’s DimPlot() function. After visualization of the UMAPs, we identified LEC clusters that expressed lymphatic endothelial markers *Prox1* and *Flt4;* blood endothelial cell clusters that expressed the blood endothelial markers *Nrp1* and *Flt1;* and immune cell clusters that expressed high levels of the immune cell marker *Ptprc*. We also observed additional clusters (in control and Kras^G12D^ datasets) that did not express the lymphatic marker *Prox1* uniformly (i.e., low percentage expression) and were composed of cells that on average had fewer numbers of genes (nFeature_RNA) and molecules (nCount_RNA) compared with the other identified clusters. These clusters may represent damaged or senescent cells and were excluded from further analysis. For downstream analysis, we only subset the LEC clusters. Additionally, we subset cells with expression levels of *Vegfr3* greater than zero (*Flt4* > 0) and levels of *Vegfr1* equal to zero (*Flt1* = 0). These filtering steps were included to ensure optimal quality and purity of LECs for further downstream analysis. After each subset step, we repeated all the steps from normalization to UMAP dimensionality reduction on control and Kras^G12D^ datasets, only adjusting the parameters for FindNeighbors(), FindClusters(), and RunUMAP(). The clusters were annotated using known LEC subtype–specific markers (21, 22).

### Integration of control and KrasG12D datasets

After loading the individual datasets and creating Seurat objects, the control and Kras^G12D^ datasets were merged using the merge() function. We then performed an initial data cleaning step to filter cells based on QC metrics as described in the previous section. The control and Kras^G12D^ datasets were batch corrected and integrated using Seurat’s data integration workflow, which involves performing canonical correlation analysis (CCA) and identifying mutual nearest neighbors (37). After integration, the Seurat workflow involving ScaleData(), RunPCA(), RunUMAP(), FindNeighbors(), and FindClusters() was performed. The clusters were visualized using the DimPlot() function. We then subset the integrated dataset to include only those clusters that expressed *Prox1* uniformly (i.e., high percentage expression) and did not contain cells with very low numbers of genes (nFeature_RNA) or low numbers of molecules (nCount_RNA). Additionally, we subset cells with expression levels of *Vegfr3* greater than zero (*Flt4* > 0) and levels of *Vegfr1* equal to zero (*Flt1* = 0). After each subset step, we re-integrated, scaled, clustered, and visualized the cells as mentioned above. The clusters were annotated using known LEC subtype–specific markers as described above in *scRNA-Seq data processing*.

### Integration of control and developmental datasets

The control and 4 developmental datasets (27) were loaded, and Seurat objects were created for each dataset. The datasets were merged, filtered based on QC metrics, integrated, and scaled and processed in a similar way as the control and Kras^G12D^ datasets. The clusters were annotated using known LEC subtype–specific markers as described above.

### DGE analysis

DGE analysis was performed using the MAST R/Bioconductor package (38). The FindAllMarkers() function in Seurat was used for determining cell type–specific markers for heatmaps, with the min.pct argument set to 0.25, logfc.threshold argument set to 0.25, and test.use argument set to “MAST.” To create a list of all differentially expressed genes in the control and integrated datasets, we used the FindAllMarkers() and FindMarkers() functions, respectively, with the min.pct argument set to 0.1, logfc.threshold argument set to 0.25, only.pos argument set to FALSE, and test.use argument set to “MAST.” The FindMarkers() function was used to obtain the differentially expressed genes for each Kras^G12D^ versus control LEC subtype.

### Trajectory inference analysis

The monocle method was used to conduct trajectory inference analysis on control and Kras^G12D^ datasets using the Monocle 3 R package (v1.3.4) (39–41). We used the UMAP coordinates, and cluster labels previously generated by Seurat by converting the Seurat object to the CDS object.

### GO analysis

GO analysis was performed using the Metascape web resource (https://metascape.org/gp/index.html#/main/step1) (42). To determine the GO terms from a list of input genes, Metascape employs the hypergeometric test and Benjamini-Hochberg *P* value correction algorithm (42). To determine the GO terms associated with the control LEC subtypes, we provided Metascape with a list of significantly upregulated genes from each subtype. For analyzing the integrated dataset, we used a list of significantly upregulated or downregulated genes in Kras^G12D^ LECs as an input for Metascape. We plotted the biologically relevant GO terms using GraphPad Prism statistical analysis software (v9.5.1).

### Capillary and collecting LEC gene signatures

To determine the capillary and collecting vessel signature scores, we used the AddModuleScore() function in Seurat. The marker genes used to calculate the capillary signature scores were *Lyve1*, *Gjc2*, *Fndc1*, *Piezo2*, *Ccl21a*, and *Ackr2*. The marker genes used to calculate the collecting signature scores were *Bgn*, *Foxp2*, *Plk2*, *Sertad1*, *Fabp4*, *Ccn1*, *Cdkn1a*, *Adamts1*, *Nos3*, *Apoe*, *Ccdc3*, *Gpx1*, *Lrg1*, and *Sgk1*. The capillary and collecting signature scores were graphed as violin plots using Seurat’s VlnPlot() function.

### Whole-mount immunofluorescent staining

We fixed ears overnight with 1% paraformaldehyde (PFA). Next, we washed the fixed samples with PBS, permeabilized them with PBS plus 0.3% Triton X-100 (PBST), and blocked them overnight with PBST plus 3% donkey serum. After that, we incubated the samples overnight with chicken anti-GFP primary antibody (Abcam, ab13970; 1:1000) diluted in PBST. Then, we washed the samples with PBST (3 times, 40 minutes each) and incubated them overnight with secondary antibodies diluted in PBST. Finally, we washed the samples with PBST (3 times, 40 minutes each), placed them on glass slides, and mounted coverslips with ProLong Gold (Invitrogen, 36934).

### Analysis of ear skin whole mounts

To assess branch points per millimeter of vessel, we measured the length of the lymphatic network and then manually counted the number of lymphatic branch points. To measure vessel diameters, we placed a 15 × 15 grid over images and measured the diameter of vessels located on intersecting grid lines. To assess lymphatic valves per millimeter of vessel, we measured the length of the lymphatic network and then manually counted the number of lymphatic valves. We used NIS Elements software (v5.30.02) to manually count branch points and valves and to measure vessel lengths and diameters.

### Lung inflation apparatus assembly

A 20 mL Luer-Lok tip syringe was attached to a ring stand 25 cm above the animal dissection platform. A stopcock was twisted into the bottom of the Leuer-Lok tip syringe, and rubber tubing was connected to the stopcock. A blunt-ended needle was coupled to an adapter joined to the rubber tubing.

### Lung inflation, sectioning, staining, and analysis

Mice were euthanized and then perfused with heparin/DPBS pH 7.2 to remove red blood cells. The blunt-ended needle joined to the lung inflation apparatus was inserted into a small incision in the trachea and secured with sutures. The stopcock was opened, allowing 4% PFA to inflate the lungs for 5 minutes. The lungs were then collected, and the suture was tied at the end of the trachea. The lungs were placed in a 50 mL conical tube containing 4% PFA, and the sutures were held in place by the threads of the cap and tube. The 50 mL conical tube was then inverted, allowing the lungs to be fully submerged while fixing overnight at 4°C. Samples were washed with 1× PBS 3 times (5 minutes each) and placed in cryoprotect solution (20% sucrose + 2% polyvinylpyrrolidone in 1× PBS) overnight at 4°C. The next day, samples were transferred to a 50 mL conical tube containing prewarmed (54°C) gelatin embedding medium (8% powdered gelatin + 20% sucrose + 2% polyvinylpyrrolidone in 1× PBS) and stored overnight at 54°C. Samples were then placed in cryomolds containing gelatin embedding medium, cooled overnight in the refrigerator, and stored in a –80°C freezer. We used a cryostat to cut 100-μm-thick sections, which we placed on glass slides. The slides were then placed in a 37°C incubator for 10 minutes. The tissues were transferred to a 24-well plate containing water. The samples were washed 3 times (5 minutes each) and then permeabilized with PBST for 1 hour at room temperature. We then followed our protocol for whole-mount immunofluorescent staining of mouse ear skin. We used a goat anti-Vegfr3 antibody (R&D Systems, AF745; 1:250). Samples were transferred to glass slides and cover slips mounted with ProLong Gold (Invitrogen, 36934). Images were captured with a 4× objective, and we used NIS Elements software (v5.30.02) to measure vessel diameters. Briefly, a 50 μm × 50 μm grid was placed over the images, and vessel diameters were measured at sites where there were intersecting gridlines.

### Analysis of tissue sections

Ear skin was fixed overnight in 4% PFA, washed with PBS (3 times, 5 minutes each), and submitted to the histology core for processing. A microtome was used to cut 5-μm-thick sections, which were then placed on microscope slides. Slides were deparaffinized with xylene and rehydrated through a descending ethanol series. Antigen retrieval was performed by heating slides in 0.01 M citric acid (pH 6.0) in a pressure cooker. Nonspecific binding was blocked by incubating slides with Tris-buffered saline with 0.2% Tween 20 (TBST) plus 3% donkey serum for 30 minutes. Slides were then incubated overnight with goat anti-Prox1 (R&D Systems, AF2727; 1:250) and rabbit anti-Lyve1 (Abcam, ab14917; 1:250) primary antibodies diluted in TBST plus 5% BSA. Slides were washed with TBST and then incubated with fluorophore-conjugated secondary antibodies diluted in TBST plus 5% BSA. Following washes with TBST, coverslips were mounted with ProLong Gold plus DAPI (Invitrogen, 36935). Four to 12 lymphatics were analyzed per sample. We used NIS Elements software (v5.30.02) to measure the length of Lyve1-positive lymphatic vessels and count the number of Prox1-positive nuclei per lymphatic vessel.

### Intralobular lymphatic vessel imaging

*Flt4CreER^T2^;Kras^+/G12D^* mice were crossed with the *Rosa26^mT/mG^* reporter line to visualize all lymphatic vessels. Tamoxifen (100 μg) was administered subcutaneously to newborn pups on P0 and P2 to activate Cre recombinase in VEGFR3-expressing cells and tissues were analyzed 3 weeks later. Mice were euthanized on P21 and whole lungs were excised from the chest cavity with the trachea and heart attached. The lungs were then placed in a dish containing a silicon bottom (made of Sylgard 170) and minuten pins were used to position the tissue with the ventral side facing up while bathed in ice-cold PBS. Images were immediately taken of the collecting lymphatic vessels entering each lobe on a Zeiss AxioZoom V16 microscope.

### Statistics

Data were analyzed using GraphPad Prism statistical analysis software (v9.5.1). A Shapiro-Wilk normality test was performed to assess whether the data were normally distributed. If the data were normally distributed, 2-tailed, unpaired Student’s *t* tests were performed to test means for significance. When the data were not normally distributed, the Mann-Whitney test (2-tailed) was conducted. All results are expressed as mean ± SEM. The number of mice in each group is indicated in the figure legends (*n* = number of mice). A *P* value of less than 0.05 was considered significant.

### Study approval

The animal experiments described in this study were carried out in accordance with an animal protocol approved by the Institutional Animal Care and Use Committee of UT Southwestern Medical Center.

### Data availability

All sequencing data have been deposited in the NCBI Gene Expression Omnibus (GEO) under accession number GSE272843 and are publicly available as of the date of publication. Raw data can be found in the Supporting Data Values file.

## Author contributions

LMF conducted experiments, acquired data, analyzed data, wrote the first draft of the manuscript, and revised the manuscript. DGW, JDT, AV, NV, and AS conducted experiments. BL analyzed data and revised the manuscript. JPS designed research studies, acquired funding, and revised the manuscript. MTD designed research studies, analyzed data, acquired funding, and wrote and revised the manuscript.

## Supplementary Material

Supplemental data

Supplemental data set 1

Supplemental data set 2

Supplemental data set 3

Supplemental data set 4

Supporting data values

## Version 1 01/28/2025

In-Press Preview

## Version 2 03/10/2025

Electronic publication
