# Supplementary material for: A single-cell atlas of normal and KRASG12D-malformed lymphatic vessels
Source: JCI Insight. 2025 Jan 28;10(5):e185181. doi: 10.1172/jci.insight.185181 (PMC11949019; doi:10.1172/jci.insight.185181)
Supplement: Supplemental data [file jciinsight-10-185181-s106.pdf]

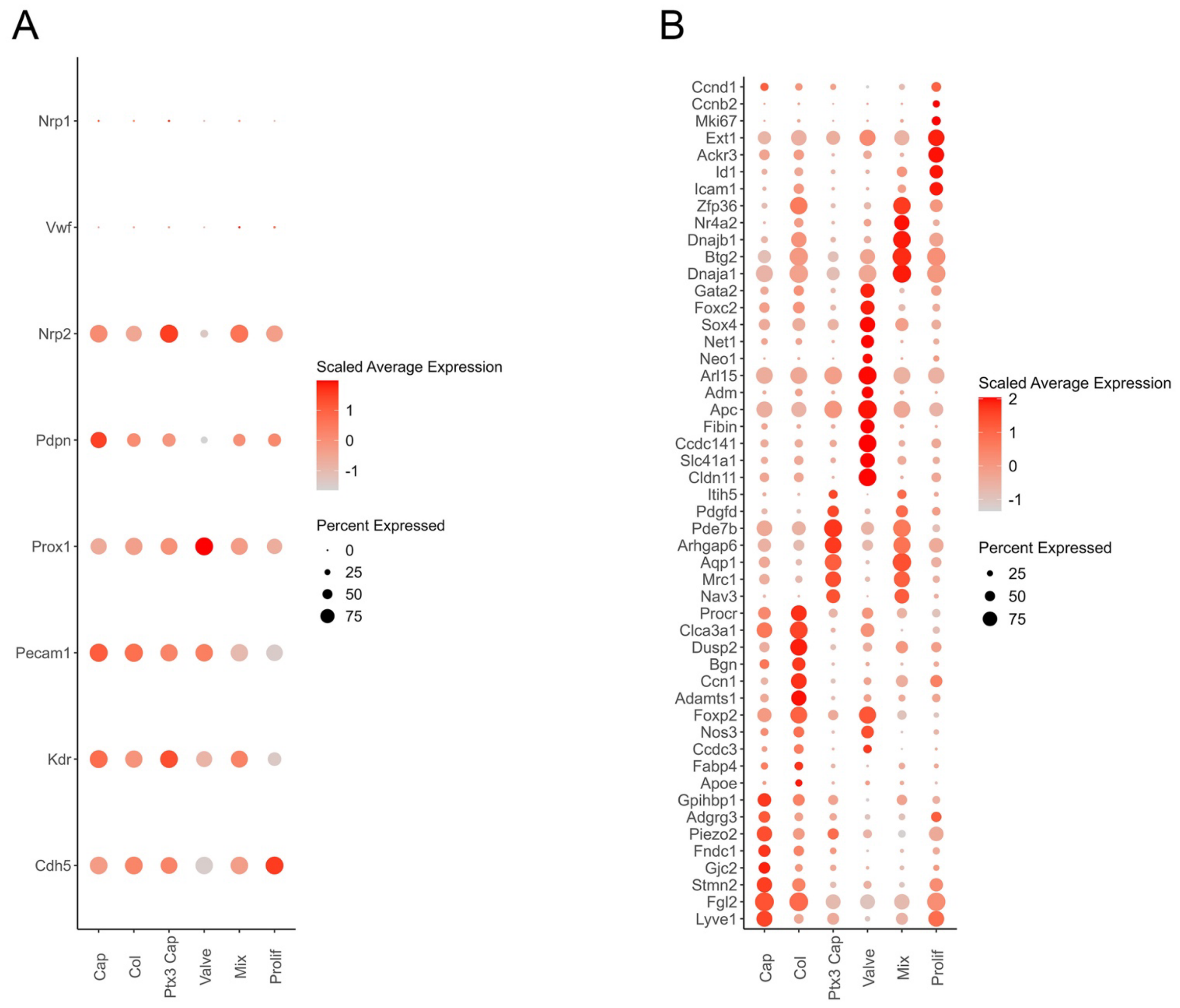

Supplemental Figure 1. LEC, BEC, and LEC-subtype markers. A. Dot plot showing markers for LECs and BECs in Control LEC clusters. The color gradient scale shows scaled average expression level in each cluster. The dot size indicates the percent of cells in each cluster expressing the given gene. **B.** Dot plot showing subtype-specific markers in Control LEC clusters. The color gradient scale shows scaled average expression level in each cluster. The dot size indicates the percent of cells in each cluster expressing the given gene.

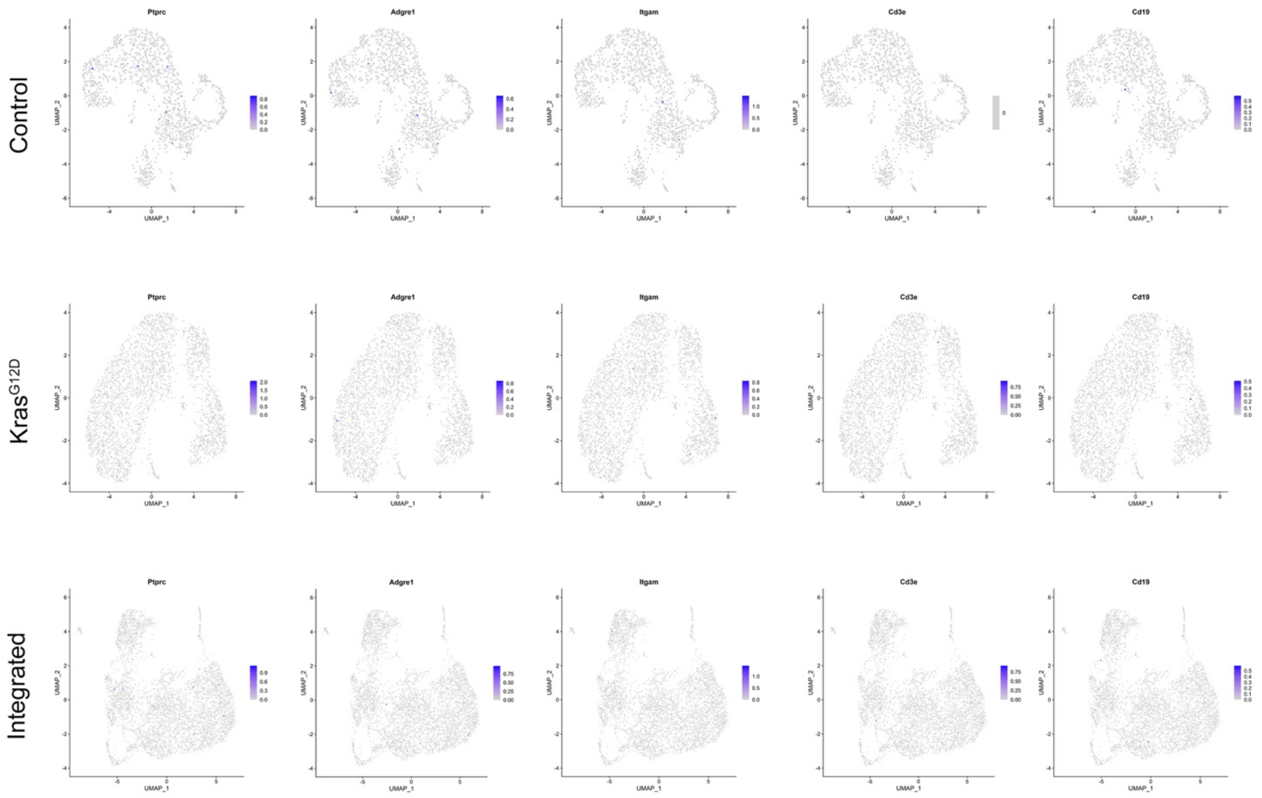

Supplemental Figure 2. UMAPs of immune cell markers. UMAPs showing expression of *Ptprc* (CD45), *Adgre1* (F4/80), *Itgam* (CD11b), *Cd3e*, and *Cd19* in our control, Kras^{G12D}, and integrated scRNA-Seq data sets.

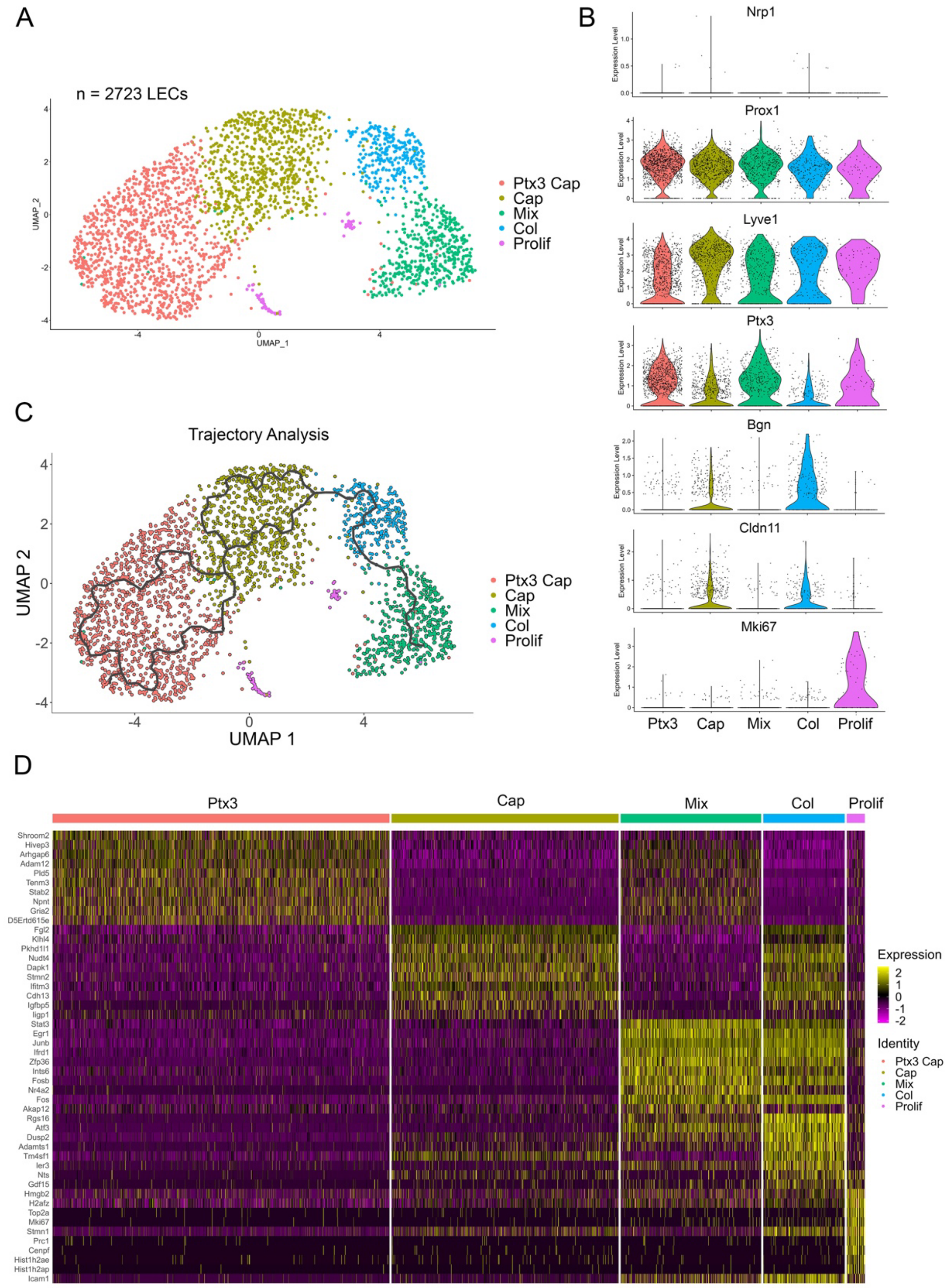

Supplemental Figure 3. scRNA-Seq results for Kras^{G12D} mice. A. UMAP showing the clustering of LECs (n = 2723 cells) from Kras^{G12D} mice. **B.** Violin plots showing the expression of select BEC and LEC markers and LEC subtype markers. **C.** Trajectory analysis of LECs from Kras^{G12D} mice using Monocle 3. **D.** Heatmap showing the top ten most differentially expressed genes for each LEC subtype.

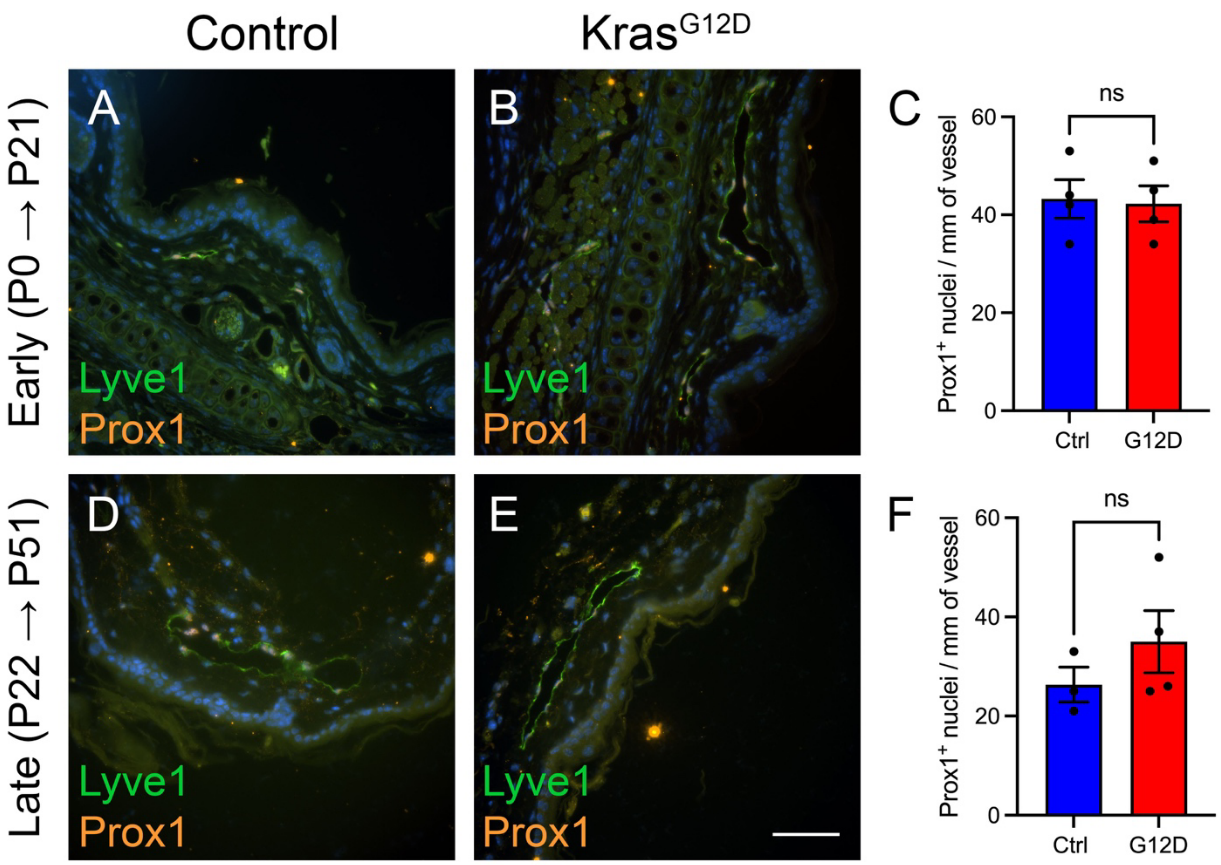

Supplemental Figure 4. Number of Prox1-positive nuclei per millimeter lymphatic vessel. A,B. Representative images of ear skin stained with antibodies against Prox1 and Lyve1. The mice received tamoxifen early. **C.** The number of Prox1-positive nuclei per millimeter of lymphatic vessel was not significantly different between control (n = 4 mice) and Kras^{G12D} mice (n = 4 mice). **D,E.** Representative images of ear skin stained with antibodies against Prox1 and Lyve1. The mice received tamoxifen late. **F.** The number of Prox1-positive nuclei per millimeter of lymphatic vessel was not significantly different between control (n = 3 mice) and Kras^{G12D} mice (n = 4 mice). ns = not significant, unpaired Student's *t*-tests. Scale bar = 50 μm.

Supplementary File 1. LEC subtype markers.

Supplementary File 2. GO term results for LEC subtypes.

Supplementary File 3. Genes differentially expressed by Kras^{G12D}.

Supplementary File 4. GO term results for genes upregulated or downregulated by Kras^{G12D}.
